# 

**Published:** 1885-08

**Authors:** 


### BOOKS AND PAMPHLETS RECEIVED.

ON THE WASTING DISEASES OF INFANTS AND CHILDREN. By Eustace Smith, M. D. London.

THE OLEATES. By Jno. V. Shoemaker, A. M., M. D. Philadelphia: F. A. Davis. 1885.

ON IDIOPATHIC ANÆMIA. By J. H. Musser, M. D. Reprint Proceedings Philadelphia Co. Medical Society. 1885.

FIFTY CASES OF ABDOMINAL SECTION, with remarks on Loparotomy—Ovariotomy. Both by J. B. Hunter, M. D. Reprints from N. Y. *Medical Journal.*

SCARLET FEVER. By T. Griswold Comstock, M. D. St. Louis: Reprint N. Y. *Medical Times.* March, 1885.

MANY DRUGS—FEW REMEDIES. By Geo. T. Welch, M. D. Reprint N. Y. *Medical Record.*

URINARY AND RENAL DERANGEMENTS AND CALCULOUS DISORDERS, Hints on Diagnosis and Treatment. By Lionel S. Beale, M. D. Philadelphia: P. Blakiston, Son & Co.

POISONS—Their Effects and Detection. By Alex. Wynter Blyth, M. R. C. S., Eng., etc. Vol. I. New York: Wm. Wood & Co. 1885.

SUERSENS OBTURATORS—Their Construction and Use. By Dr. Th. Weber Helsingfor Finland. Reprint *Independent Practitioner.* April, May and June. 1885.

BACTERIAL PATHOLOGY. By Watson Cheyne. London: New York Industrial Publishing Company. 1885:

ELEVENTH ANNOUNCEMENT AMERICAN VETERINARY COLLEGE. 1885.

Hay Fever and its Successful Treatment. By Chas. E. Sajous, M. D. Philadelphia: F. A. Davis, Atty. 1885.

A Treatise on Asiatic Cholera. Edited and prepared by Edmund Charles Wendt, M. D. New York: Wm. Wood & Co. 1885.

Epidemic of Typhoid Fever, at Plymouth, Pa. Reprint from Proceedings Philadelphia Co. Medical Society. May 1, 1885.

Medical Legislation—The Annual Address, delivered before the Association of American Medical Editors. By Henry O. Marcy, M. D. Boston, Mass.: Reprint *Journal American Medical Association.* May 2, 1885.

An Act to establish a Board of Registration in Medicine and Surgery, from Massachusetts.

Constitutional Treatment of Caries and Necrosis. By Hal C. Wyman, M. D. Detroit, Mich.

An Accidental Divulsion of a Pteryguem, Leading to an Improvement in the Regular Operation. By A. E. Prince, M. D. Jacksonville, Ill.: Reprint *Archives of Ophthalmology.*

The Pulley Modification of His Limiting Tenatomy and Advancement of the Rectus Operation, with Cases. By A. E. Prince, M. D. Jacksonville, Ill.: Reprint *Medical Record.*

Floating Minute Organic Matter in the Air, and its Management to Prevent Disease and to Mitigate or Control It, With a New Device for Atmospheric Purification. By D. Prince, M. D. Jacksonville, Ill.: Reprint St. Louis *Medical and Surgical Journal.*

Medical Society of the State of Tennessee. Transactions 1885. C. C. Fite, M. D., Secretary. Nashville.

Mission and Methods of Medicine. An Address delivered by R. H. Day, M. D., President of the Louisiana State Medical Society, at its Seventh Annual Session. New Orleans: April 21, 1885.

